# Rapid determination of paeoniflorin

**DOI:** 10.4103/0973-1296.71781

**Published:** 2010

**Authors:** Viroj Wiwanitkit

**Affiliations:** *Wiwanitkit House, Bangkhae, Bangkok, Thailand 10160*

Sir,

I read the recent publication on rapid determination of paeoniflorin with great interest.[1] Zhou *et al*. concluded that their new proposed method is a rapid, effective, binary reverse phase rapid resolution liquid chromatographic (LC) method.[1] I have some comments on this work. First, I have a concern on the cost of this new LC method. Is it expensive that prevents it from application in routine usage? Second, the quality management, especially for external quality assessment, is a problem that might prevent the generalization of this new technique.[2] Third, the LC is not the fastest technique for identification. Fluorescence- and electrochemical-based detections might be faster.
